# Utilization of In-Hospital Care among Foreign-Born Compared to Native Swedes 1987–1999

**DOI:** 10.1155/2012/713249

**Published:** 2012-10-31

**Authors:** Björn Albin, Katarina Hjelm, Jan Ekberg, Sölve Elmståhl

**Affiliations:** ^1^School of Health and Caring Sciences, Linnaeus University, 35 195 Växjö, Sweden; ^2^Division of Geriatric Medicine, Department of Health Sciences, Lund University, 22 100 Lund, Sweden; ^3^Centre of Labour Market Policy Research (CAFO), School of Management and Economics, Linnaeus University Sweden, 35 195 Växjö, Sweden

## Abstract

In previous longitudinal studies of mortality and morbidity among foreign-born and native-born Swedes, increased mortality and dissimilarities in mortality pattern were found. The aim of this study is to describe, compare, and analyse the utilization of in-hospital care among deceased foreign- and Swedish-born persons during the years 1987–1999 with focus on four diagnostic categories. The study population consisted of 361,974 foreign-born persons aged 16 years and upward who were registered as living in Sweden in 1970, together with 361,974 matched Swedish controls for each person. Data from Statistics Sweden (SCB) and the National Board of Health and Welfare Centre for Epidemiology, covering the period 1970–1999, was used. Persons were selected if they were admitted to hospital during 1987–1999 and the cause of death was in one of four ICD groups. The results indicate a tendency towards less health care utilization among migrants, especially men, as regards *Symptoms*, *signs,* and *ill-defined conditions* and *Injury* and *poisoning*. Further studies are needed to explore the possible explanations and the pattern of other diseases to see whether migrants, and especially migrant men, are a risk group with less utilization of health care.

## 1. Introduction

In previous longitudinal studies of mortality and morbidity among 723,948 foreign-born and native-born Swedes during 1970–1999, increased mortality and dissimilarities in mortality pattern were found [1, 2]. The results showed increased mortality (OR = 1.08, 95% CI: 1.07–1.08) and a lower age at time of death for foreign-born persons compared with the Swedish controls. The highest risk odds were found for men born in Finland (OR = 1.21), Denmark (OR = 1.11), and Norway/Iceland (OR = 1.074). The mortality pattern showed dissimilarities in causes of death, with a significant higher number of deaths from* Neoplasm* found in migrants from Denmark in, from* diseases of the circulatory system* in migrants from Finland and Poland and from* symptoms, signs, and ill-defined conditions* in migrants from former Yugoslavia.

The differences in mortality and morbidity pattern could indicate differences in utilization of health care among foreign and native-born Swedes; this needs to be further studied. 

Earlier studies of utilization of health care among immigrants and the native population have had a predominantly cross-sectional design and have shown diverging results. Lower utilization has been explained as lack of economic resources such as access to health insurance, and the higher utilization could be a consequence of poorer health [3–10]. Results from The Netherlands showed differences between different migrant groups with more use of hospital care among persons from The Netherlands Antilles and more use of care from a general practitioner among persons from Surinam, Turkey, and Morocco [11]. In Denmark, two studies found no differences for time in hospital between migrants and nonmigrants [12] but persons from Somalia, Turkey, and former Yugoslavia had higher utilization rates for emergency room than Danish-born persons. This was explained by disparities in health, lack of knowledge about the health care system or low satisfaction with primary care [13]. Studies in Sweden have found that migrants from Chile, Iran, and Turkey had more visits to a physician during a three-month period than native Swedes [14]. This was due to a lower self-rated health and previous exposure to violence in their country of origin. One longitudinal study of admission to hospital for women during 1993–1998 showed a higher risk of psychiatric hospital admission for all foreign-born, but only non-European refugees had a higher risk of somatic hospital admission [15]. In a Swedish study, health care expenditure was not seen as related to country of birth; instead, individual low income and living alone explained the differences demonstrated [16]. Previous predominantly cross-sectional studies of utilization of health care among immigrants and the native population have shown diverging results, and a systematic literature review found a lack of appropriate epidemiological data [16]. This justified new studies, as this one, with a longitudinal design.

Foreign-born persons living in Sweden today constitute 11.5 percent of the total Swedish population, or slightly more than 1 million persons [17]. The migrant population is dominated by labour migrants from the Nordic countries, especially Finland, and European countries like Yugoslavia, Germany, and Poland, but the whole migrant population represents about 140 different nationalities [17].

The aim of this study is to describe, compare, and analyse the utilization of health care, measured as the number of hospital admissions and days in hospital, among deceased foreign and Swedish-born persons during the years 1987–1999. The study will focus on four diagnostic categories where significant differences in mortality and morbidity have been shown. The pattern will be discussed in relation to gender, year of birth, and country of birth testing the hypothesis that no differences exist in health care utilization in relation to being foreign-born. 

## 2. Material and Methods

This study is a case-control study based on national register data from a large number of subjects. The study population consisted of 361,974 foreign-born persons aged 16 years old and upward who were registered as living in Sweden in 1970, together with 361,974 matched Swedish controls for each person. This database was originally set up by CAFO (Centre for Labour Market Research) at Växjö University. The data came from Statistics Sweden (SCB) and the National Board of Health and Welfare Centre for Epidemiology, covering the period 1970–1999 and including all foreign-born persons registered as living in Sweden in 1970. The control was matched and was similar in age (±3 year), sex, occupation, and type of employment and lived in the same county in 1970. Type of employment was divided into three groups (government, municipal, or other employer). Occupation was coded according to the Nordic Occupation Classification System (NYK), and county represented all the 24 county council areas in Sweden. This data relates to the situation on November 1, 1970 and was taken from the National Census of 1970, which was a total census, and checked against the National Population Register (RTB), which included data up to December 31, 1999. Each person was given a code if, he/she was deceased, still living in Sweden, had emigrated, or if no information was available. Information from the National Board of Health and Welfare Centre for Epidemiology on date of death and death diagnosis was added to the database. In total, 906,564 people were included with 50 percent foreign-born persons.

 A Swedish matched control could not be found for 20,518 of the foreign-born persons due to the matching criteria. 

Exclusion criteria were if no information was available or if a person had emigrated or migrated back (“remigrated”); thus, in total 163,896 persons were excluded from the database. Persons were then also excluded if the information from the control subject was missing due to migration. The database used for analysis finally consisted of 723,948 persons. Causes of death were registered according to the system of International Classification of Diseases (ICD) revision 8 (1969), 9 (1987), or 10 (1998). 

From the database, persons were selected if their registered causes of death were in one of the ICD groups of *Neoplasms *(code 140-239 and C00-D48), *Diseases of the circulatory system* (code 390-459 and I00-I99), *Injury and poisoning* (code 800-999 and S00-T98), or *Symptoms, signs, and ill-defined conditions* (code 780-799 and R00-R99). They also had to be admitted to hospital care at least one time during the studied time period 1987–1999.

 The rationale for studying the selected ICD groups was an earlier study showing significant higher numbers of deaths among foreign-born than native Swedes in these groups.

The analysis involved, first, a comparison of foreign-born with a selected cause of death with Swedish-born persons with the same cause of death and secondly a comparison of groups of foreign-born from specific countries or regions with the total group of Swedish-born with the same cause of death. Natives from the following countries have been studied in particular: Denmark, Finland, Norway/Iceland, Yugoslavia, Poland, Germany, other European countries, and non-European countries. The rationale for studying the selected countries was that increased mortality and different pattern of causes of death had been shown among these migrant groups in previous analyses and that they constitute the dominant groups (74.9%) of all migrants in Sweden included in the database during the studied period.

### 2.1. Statistical Analysis

Values are given as numbers, means, and percentages. Comparisons were made by tests of significance with Mann-Whitney *U*-test. A value of *P* < 0.05 was considered statistically significant [18]. Stepwise multiple-linear regression was performed with age at death, being foreign-born, and age as independent variables to investigate the importance of them on the dependent variables, number of hospital admissions, total number of days in hospital, and number of days in hospital during the last two hospital admissions. 

All analyses were performed using SPSS (Statistical Package for Social Sciences), version 11,5. 

### 2.2. Ethics

The Ethics Committee of the University of Lund approved the study after all other Swedish University Ethics Committees had reviewed it. 

## 3. Results

### 3.1. Characteristics of the Study Population

The total number of deceased foreign-born persons was 41,688 persons, of whom 15,372 had *Neoplasms *as cause of death and 23,837 persons *Diseases of the circulatory system* and 1605 persons were related to the death diagnosis *Injury and poisoning* and 874 persons had *Symptoms, signs, and ill-defined conditions* as cause of death. Corresponding numbers for Swedish-born were 44,941 persons in total, of whom 16,651 had *Neoplasms* and 26,042 had *Diseases of the circulatory system* as cause of death. Persons with *Injury and poisoning* and with *Symptoms, signs, and ill-defined conditions* as cause of death were 1671 and 577 respectively (Table 1).

### 3.2. The Number of Days in Hospital during the Last Two Hospital Admissions

The number of days in hospital during the two last hospital admissions was similar for foreign-born and native Swedes for persons with *Neoplasm* as cause of death. Foreign-born persons with *Diseases of the circulatory system* as cause of death had a higher number of days than native Swedes (48.2 versus 46.0 *P* = 0.001). Foreign-born persons had a higher number of days (32.1 versus 29.9, *P* < 0.001) than Swedish-born persons in the ICD group *Injury and poisoning* but a lower number of days in the ICD group *Symptoms, signs and ill-defined conditions* (55.5 versus 78.2, *P* < 0.001), see Table 1.

Multiple-linear regression analysis, with age and being foreign-born as independent variables, showed that being a foreign-born person was a significant factor in relation to number of days in hospital during the two last hospital admissions only in the ICD diagnosis group *Diseases of the circulatory system* (*P* = 0.005, *β* = 0.012), Table 2.

### 3.3. Number of Hospital Admissions

The total number of hospital admissions during 1987–1999 was similar for foreign-born and native Swedes with the two exceptions of persons who had *Injury and poisoning* and *Symptoms, signs and ill-defined conditions* as causes of death. Foreign-born had a lower number of admissions to hospital (4.7 versus 5.3, *P* = 0.041, and 5.4 versus 6.1, *P* < 0.001) than Swedish-born persons), Table 1. 

Multiple-linear regression analysis showed that being a foreign-born person adjusted for age was an independent determining factor for a higher number of hospital admissions in the ICD diagnosis groups *Diseases of the circulatory system* (*P* = 0.038, *β* = 0.009) and lower number in *Injury and poisoning* (*P* = 0.012, *β* = − 0.043) and *Symptoms, signs, and ill-defined conditions* (*P* = 0.006, *β* = − 0.072), Table 2.

### 3.4. Total Number of Days in Hospital

Total number of days in hospital differed between foreign- and Swedish-born persons in three ICD groups: *Diseases of the circulatory system*, *Symptoms, signs, and ill-defined conditions *and *Injury and poisoning*. Foreign-born had a significantly higher total number of days in hospital in the ICD group *Diseases of the circulatory system* than Swedish-born persons (114.8 versus 110.4, *P* = 0.008) but a significantly fewer number of days in hospital in the ICD groups *Symptoms, signs, and ill-defined conditions* (107.6 versus 158.6, *P* < 0.001) and *Injury and poisoning* (90.5 versus 91.3, *P* < 0.001), see Table 1.

Being a foreign-born person was an independent determining factor for the total number of days in hospital among persons with *Diseases of the circulatory system* and *Symptoms, signs, and ill-defined conditions* as cause of death. Age was also a significant factor and had a higher *β*-value in all cases, see Table 2.

### 3.5. The Influence of Gender

An analysis was also performed of the number of days during the last two admissions to hospital with regard to gender and cause of death classified as ICD groups, see Table 3. For persons with *Neoplasm* as cause of death, no differences was found between Swedish and foreign-born persons. Foreign-born men with *Diseases of the circulatory system* and *Symptoms, signs, and ill-defined conditions* as cause of death had fewer days in hospital (33.4 versus 34.8, *P* < 0.001, and 26.3 versus 41.0, *P* = 0.001) than Swedish-born men. A higher number of days in hospital was found for foreign-born men with *Injury and poisoning* as cause of death (27.2 versus 26.2, *P* < 0.001). Foreign-born women had a higher number of days in hospital than Swedish-born women (38.9 versus 35.3, *P* = 0.001) in the ICD group of *Injury and poisoning* and a lower number of days (83.3 versus 100.1, *P* = 0.014) in the ICD group *Symptoms, signs, and ill-defined conditions*, see Table 3.

In relation to gender, foreign-born men had fewer hospital admissions (4.5 versus 5.4, *P* = 0.024) for *Injury and poisoning* and *Symptoms, signs, and ill-defined conditions* (5.3 versus 6.5, *P* = 0.003), Table 4. Foreign-born women had fewer hospital admissions in relation to the diagnosis group *Symptoms, signs, and ill-defined conditions* than Swedish-born women (5.5 versus 5.9, *P* = 0.034).

When multiple linear regression analysis was performed separately for men and women, it was found that being a foreign-born person was an independent determining factor for hospital admissions adjusted for age for men in the ICD groups *Symptoms, signs, and ill-defined conditions* (*P* = 0.010, *β* = − 0.102) and *Injury and poisoning* (*P* = 0.004, *β* = − 0.065) but had less influence than age. 

### 3.6. The Influence of Age

Analysis of the number of days during the last two admissions to hospital in relation to age revealed a clear tendency, with increasing number of days in hospital with increasing age. A separate analysis of three death age intervals (25–44, 45–64, and 65–84 years) during the period 1992–1999 showed significantly fewer days in hospital in the age interval 65–84 years for foreign-born persons with *Injury and poisoning* (*P* = 0.011) and *Symptoms, signs, and ill-defined conditions *(*P* = 0.015) as cause of death, Table 5. Foreign-born persons in the age interval 45–64 years had significantly fewer days in the ICD group *Diseases of the circulatory system* (*P* = 0.026), see Table 5.

### 3.7. The Influence of Country/Region of Birth

Country/region of birth was not an independent determining factor for number of days in hospital during the two last hospital admissions when multiple-linear regression analysis was performed. Age was an independent determining factor for number of days during the two last hospital admissions in the ICD group *Symptoms, signs, and ill-defined conditions* but not in the group *Injury and poisoning*.

## 4. Discussion

The main finding in the study was the differences in utilization of hospital care between foreign-born and Swedish-born persons in three out of four selected ICD diagnosis groups.

No differences were found for persons with *Neoplasm* as cause of death. Foreign-born persons dying of *Diseases of the circulatory system* had a higher number of days in hospital, both in total and during the two last admissions, than native Swedes. Fewer days were found for foreign-born persons dying of *Symptoms, signs, and ill-defined conditions*. Foreign-born men with death diagnoses *Injury and poisoning* and *Symptoms, signs, and ill-defined conditions* had fewer hospital admissions than Swedish men.

Differences in days in hospital during the two last admissionswas not influenced by specific country/region of birth.

The similarities in number of admissions, total number of days, and number of days in hospital during the last two admissions, especially in diagnosis groups *Neoplasms* and *Diseases of the circulatory system*, could be interpreted as showing that standardized treatment and care plans are established and used for diseases related to these diagnosis groups. National guidelines and evaluations of treatment have been published for both cardiac and cancer care [19, 20].

An earlier study in Sweden of equity in access to health care for migrants has not indicated any gross pattern of inequity in the utilization of health care [14]. A study of diabetic patients showed no differences between foreign and Swedish-born persons in attendance rate to specialists in internal medicine [21]. The finding is also in accordance with the main goal, care on equal terms for the whole population stated in the Swedish Health and Medical Services Act [22].

The studied foreign-born population was mainly persons from the Nordic countries and Europe with a long stay in Sweden and labour migrant background, but it also included refugees. Adaptation to Swedish society and hence adaptation to the same utilization of health care could also explain similarities between foreign- and Swedish-born persons [21].

The number of days during the last two admissions and thetotal number of days in hospital was higher among foreign-born persons than native Swedes in the ICD group *Diseases of the circulatory system*. An explanation could be that migrants express health and illness differently from native Swedes [23] and/or difficulties in communication between patients and hospital staff have occurred [24] and made it more difficult and more time consuming to use guidelines and standardized treatment. 

Migrants showed shorter total time in hospital and for migrant men also fewer admissions to hospital in the ICD diagnosis group *Symptoms, signs, and ill-defined conditions*. The diagnosis group in itself indicates difficulties in finding a correct diagnosis, and shorter time and fewer admissions could be due to migrants' expressing health and illness differently from native Swedes [23]. Difficulties in communication have also been described as a problem among native Swedish health care staff in their relations with migrants [24], and this could also influence both admissions to hospital and number of days in hospital. The seriousness of the health problems could be underestimated due to lack of information and hence the time in hospital could be shorter or the patient might not be admitted to hospital.

Fewer days in hospital could also be explained if more care among migrants than native Swedes was carried out within the families or close social networks, but this study does not allow for this type of analysis. However, immigrant families have been described as providers of more care, especially for the aged [25], but analysis of spousal earnings and participation in the labour market among migrant households with persons with illness in the household has not found that persons leave their work to take care of family members [26]. Studies of diabetes patients in Sweden have indicated less use of home care from public authorities by foreign-born persons [21]. 

Gender differences could explain why foreign-born men but not women had fewer hospital admissions. Gender differences have been discussed, such as that more women have more contacts with physicians, more ability to recall minor health problems, and give more details about symptoms [27, 28]. Women have been described as not only more demanding but also easier to communicate with [29]. Women have also been shown to have a more active and information-searching behaviour than men [30]. Foreign-born women may thus compensate for communication problems better than foreign-born men.

Fewer hospital admissions were also found among foreign-born men in the ICD group *Injury and poisoning*. This might, as previously discussed, be a consequence of gender differences and communication problems [24, 27, 28]. The number of hospital admissions does not reflect the situation in the labour market. The working environment for migrants was known to be different from that of native Swedes, with more physically demanding and stressful work [31], which could involve greater risk of injuries. In relation to work environment women had more total injury reports than men [32], but serious accidents were more common among men [33]. Injuries were also found as the main reason for hospitalisation among younger men in a study from Italy [34].

In two ICD diagnosis groups, *Symptoms, signs, and ill-defined conditions* and *Injury and poisoning*, migrants showed significantly fewer days in hospital during the two latest hospital admissions in the age intervals 65–84 years than native Swedes. This could be a consequence of old age accentuating communication problems such as language skills among migrants [35, 36].

Previous studies have shown increased mortality and a lower mean age at time of death among foreign-born persons living in Sweden than among native Swedes [1, 2]. The analysis of utilization of hospital care, measured as total time in hospital, number of hospital admissions, and time in hospital during the last two admissions, gave no information that could explain these differences to any great extent. 

The study of utilization of hospital care among deceased foreign-born and native Swedes 1987–1999 in four selected causes of death was based on data from Statistics Sweden and from the National Board of Health and Welfare Centre for Epidemiology. Causes of death were registered according to the system of International Classification of Diseases (ICD) revision 9 (1997) or 10 (1998).

The four selected cause-of-death groups—*Neoplasms*, *Diseases of the circulatory system*, *Injury and poisoning* and *Symptoms, signs, and ill-defined conditions*—were chosen since previous studies had shown significant differences in mortality and morbidity in these groups between foreign-born persons and native Swedes [1, 2].

The time period for the study is limited to 12 years (1987–1999) because no data on hospital care was nationally registered before 1987. One important change has been made in the Swedish health care system during this period that can influence the number of total days and days during the two last admissions in hospital. Before 1992 the days elderly persons spent in nursing homes was registered as days in hospital. In 1992 the responsibility for elderly care was transferred from the counties to the local authorities and care in a nursing home was no longer regarded as hospital care. Due to this change the analysis of days during the two last admissions in hospital stratified for age was limited to the period 1992–1999. There are still no national data available in Sweden on care outside of hospital settings such as visits to GP or visits to nurses in primary care centres. 

The data used to establish the database originated from the Population and Housing Census of 1970, which is considered to be a total census as it was compulsory by law to take part in the census. No number of dropouts has been estimated for the total census, only for some of the variables such as “occupation,” and Statistics Sweden estimates the dropout on this variable to be 3.5–4.5%. It can only be speculated whether participation in the census is related to health problems and whether there were a number of migrants that did not take part as well as a number of Swedes. Other reasons for migrants not participating in the census could be language problems.

Persons were excluded from the database if they had emigrated, migrated back (remigrated), or if the control subject had migrated or if no information about the person was available. A followup of Finns who had remigrated was performed and showed no differences in mortality from the group of Finns included in the study [1]. There are no reasons to believe that “remigrants” born in other countries differ from the Finnish group.

The diagnosis given at death is in most cases not based on autopsy. The autopsy frequency in Sweden has varied during the period and has decreased from 41% for men and 31% for women (1987) to 22% for men and 13% (1998) (The National Board of Health and Welfare, 1998). There could be a bias in diagnosing the right cause death because of a low autopsy frequency. Studies of cancer incidence indicate about 10% higher incidence when autopsy is performed [35]. Another bias could occur when the correct death diagnosis code written in the register; in 1998 this *coding error *was estimated at 1.2 ± 0.2% [36].

In conclusion, the study indicates a tendency towards less health care utilization among migrants, especially men, as regards *Symptoms, signs, and ill-defined conditions* and *Injury and poisoning. *Further studies are needed to explore possible explanations and pattern of other diseases to see whether migrants, and especially migrant men, are a risk group with less utilization of health care. If the findings of less health care utilization are verified in other studies, special policy and special care plans need be developed to reduce the problems of finding the correct diagnoses and delay of correct management and, thus, also reduce the risk of premature death. 

## Figures and Tables

**Table 1:** Number of hospital admissions and days in hospital (mean values) for deceased persons 1987–1999 in four ICD groups.

Diagnosis	Foreign-born					Swedish-born				
	In-patient record	Age 1970 (95% CI)	Hospital admissions (95% CI)	Total days in hospital (95% CI)	Days during the two last hospital admissions (95% CI)	In patient record	Age 1970 (95% CI)	Hospital admissions (95% CI)	Days in hospital (95% CI)	Days during the two last hospital admissions (95% CI)
*Neoplasm *	15 372	46.5** (46.7, 47.0)	7.2 (7.1–7.3)	81.0 (78.8, 83.3)	33.1 (32.2, 33.9)	16 651	46.9 (46.7, 47.0)	7.2 (7.1–7.3)	84.1 (80.7, 87.5)	33.1 (32.2, 34.1)

*Diseases of the circ*. *syst. *	23 837	53.2* (53.9, 54.2)	6.0 (5.9–6.1)	114.8*** (110.4, 119.3)	48.2** (46.3, 50.2)	26 042	54.1 (53.9, 54.2)	5.9 (5.9–6.0)	110.4 (106.6, 114.2)	46.0 (44.3, 47.8)

*Injury and poisoning *	1605	41.7* (41.0, 42.5)	4.7**** (4.4–5.0)	90.5* (76.5, 104.4)	32.1* (25.8, 38.4)	1671	44.3 (43.6, 45.0)	5.3 (5.0–5.6)	91.3 (79.8, 102.8)	29.9 (26.3, 33.5)

*Signs and ill-defined conditions *	874	53.0* (52.0, 53.9)	5.4* (5.0–5.8)	107.6* (89.7, 125.5)	55.5* (43.6, 67.4)	577	58.2 (57.1, 59.3)	6.1 (5.5–6.62)	158.6 (124.9, 192.3)	78.2 (60.9, 95.5)

*P = 0.000, ^†∗∗^ *P* = 0.001, ^‡∗∗∗^ *P* = 0.008, ****P = 0.041.

**Table 2:** Multiple linear regression with age at death (constant) and being/not being a foreign-born person adjusted for age in relation to utilization of hospital care.

Diagnosis	Number of hospital admissions		Total number of days		Number of days at the two last hospital admissions	
	Significance	*β*-value	Significance	*β*-value	Significance	*β*-value
*Neoplasm *						
Age	0.000	−0.613	NS	—	0.000	0.864
Foreign-born person or not	NS	—	NS	—	NS	—

*Diseases of the circ*. *syst. *						
Age	0.000	−0.782	0.000	0.058	0.000	0.299
Foreign-born person or not	0.038	0.009	0.018	0.011	0.005	0.012

*Injury and poisoning *						
Age	0.000	−1.199	NS	—	NS	—
Foreign-born person or not	0.012	−0.043	NS	—	NS	—

*Signs and ill-defined conditions *						
Age	0.000	−0.718	0.000	0.677	0.000	0.898
Foreign-born person or not	0.006	−0.072	0.040	−0.054	NS	—

**Table 3:** Number of days in hospital (mean) at the two latest admissions to a hospital for deceased persons 1987–1999 in four ICD groups stratified for gender.

	Foreign-born men		wedish-born men			Foreign-born women		Swedish-born women		
Diagnosis					*P* value					*P* value
	*n*	Days in hospital (range)	*n*	Days in hospital (range)		*n*	Days in hospital (range)	*n*	Days in hospital (range)	
*Neoplasm *	7646	30.6 (0, 1438)	8424	30.8 (0, 1305)	0.313	7726	35.5 (0, 1625)	8227	35.6 (0, 3211)	0.362
*Diseases of the circ*. *syst. *	11 614	33.4 (0, 2193)	13 156	34.8 (0, 1980)	0.000	12 222	62.3 (0, 2532)	12 886	57.5 (0, 2253)	0.297
*Injury and poisoning *	933	27.2 (0, 2474)	996	26.2 (0, 1091)	0.000	672	38.9 (0, 1632)	675	35.3 (0, 808)	0.001
*Signs and ill-defined conditions *	426	26.3 (0, 1539)	214	41.0 (0, 1671)	0.001	448	83.3 (0, 1775)	362	100.1 (0, 1673)	0.014

**Table 4:** Number of hospital admissions (mean) in relation to gender for deceased persons 1987–1999 in four ICD groups.

Diagnosis	Foreign-born men			Swedish-born men				Foreign-born women			Swedish-born women			
	*n*	Age 1970 (95% CI)	Admissions (range)	*n*	Age 1970 (95% CI)	Admissions (range)	*P* value	*n*	Age 1970 (95% CI)	Admissions (range)	*n*	Age 1970 (95% CI)	Admissions (range)	*P* value
*Neoplasm *	7646	46.0* (45.8, 46.2)	7.0 (1, 115)	8424	47.8 (46.6, 47.0)	7.0 (1, 134)	0.717	7726	47.0 (46.8, 47.3)	7.4 (1, 125)	8227	47.0 (46.7, 47.2)	7.3 (1, 79)	0.488

*Diseases of the circ*. *syst. *	11 615	49.6* (49.4, 49.8)	6.0 (1, 107)	13 156	51.0 (50.8, 51.2)	6.0 (1, 99)	0.776	12 753	56.6* (56.4, 56.8)	5.9 (1, 114)	13 426	57.2 (57.0, 57.4)	5.8 (1, 107)	0.746

*Injury and poisoning *	933	38.5* (37.6, 39.4)	4.5 (1, 96)	996	41.0 (40.1, 41.8)	5.4 (1, 95)	0.024	672	46.1** (44.9, 47.4)	4.9 (1, 74)	675	49.3 (48.0, 50.5)	5.1 (1, 52)	0.641

*Signs and ill-defined conditions *	426	46.1* (44.9, 47.4)	5.3 (1, 60)	214	51.5 (49.5, 53.6)	6.5 (1, 76)	0.003	448	59.5*** (58.4, 60.6)	5.5 (1, 52)	363	62.1 (61.1, 63.2)	5.9 (1, 55)	0.034

*P = 0.000, **P = 0.001, ***P = 0.003.

**Table 5:** Days in hospital during the two latest admissions stratified for age groups among foreign and Swedish-born persons with deceased in four different diagnose groups 1992–1999.

Age (years)	Foreign-born			Swedish-born			*P* value
	*n*	Days in hospital mean	Range	*n*	Days in hospital mean	Range
*Neoplasm *							
65–84	6294	28.8	0, 1625	7011	28.7	0, 1305	0.889
45–64	2849	26.9	0, 528	2880	26.6	0, 902	0.621
25–44	73	26.2	2, 106	71	33.5	1, 873	0.305

*Diseases of the circulatory system *							
65–84	9588	35.6	0, 2532	10 636	34.7	0, 2253	0.265
45–64	2184	19.4	0, 1477	1882	17.3	0, 1254	0.026
25–44	45	12.5	0, 188	27	15.3	0, 143	0.903

*Injury and poisoning *							
65–84	283	32.3	0, 2054	349	32.4	0, 832	0.011
45–64	365	24.8	0, 2474	354	25.5	0, 1091	0.509
25–44	71	39.7	0, 1738	47	17.1	1, 159	0.630

*Signs and ill-defined conditions *							
65–84	275	26.5	0, 922	109	32.7	1, 539	0.015
45–64	144	13.2	0, 136	64	39.8	0, 1671	0.307
25–44	2	8.5	7, 10	2	8.0	4, 12	1.00
